# Supplementary material for: Deletion of Trim28 in committed adipocytes promotes obesity but preserves glucose tolerance
Source: Nat Commun. 2021 Jan 4;12:74. doi: 10.1038/s41467-020-20434-3 (PMC7782476; doi:10.1038/s41467-020-20434-3)
Supplement: Supplementary file 1 — Supplementary Information [file 41467_2020_20434_MOESM1_ESM.pdf]

Deletion of Trim28 in Committed Adipocytes Promotes Obesity but Preserves Glucose Tolerance

Simon T. Bond, Emily J. King, Darren C. Henstridge, Adrian Tran, Sarah C. Moody, Christine Yang, Yingying Liu, Natalie A. Mellett, Artika P. Nath, Michael Inouye, Elizabeth J. Tarling, Thomas Q. de Aguiar Vallim, Peter J. Meikle, Anna C. Calkin & Brian G. Drew

Supplementary Information.

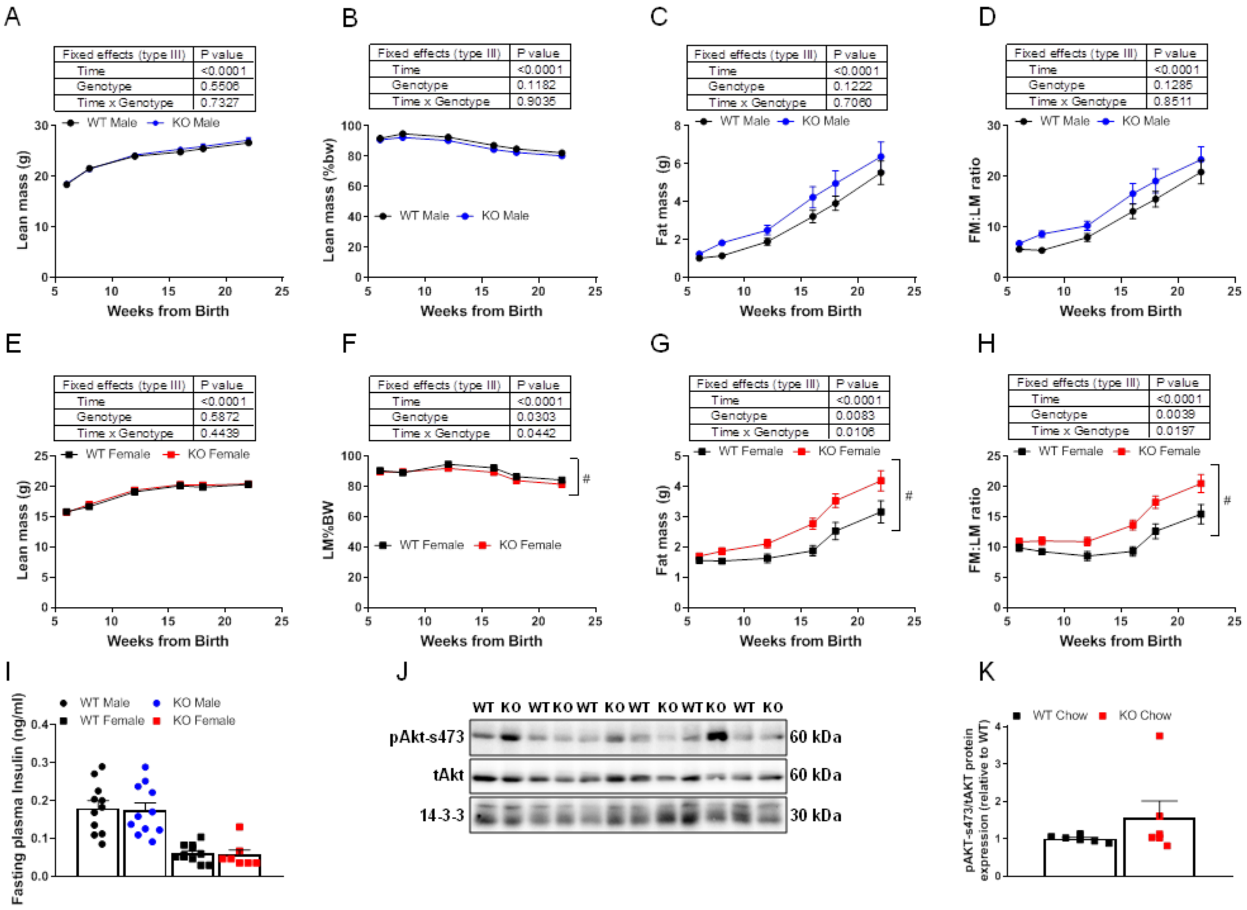

Supplementary Figure 1: Body composition and metabolic parameters in chow fed mice. Lean mass in male mice (A) in grams, and (B) lean mass as a percent of bodyweight (%BW). Fat mass in male mice (C) in grams and (D) fat mass to lean mass ratio (FM:LM). Lean mass in female mice (E) in grams, and (F) lean mass as a percent of bodyweight. Fat mass in female mice (G) in grams and (H) fat mass to lean mass ratio. (I) Fasting plasma insulin levels and (J) Western blot for phosphorylated Akt at serine 473 (pAkt-s473), total Akt (tAkt) and 14-3-3 in livers of chow fed WT and Trim28 adi-KO female mice (K) Quantification of Akt phosphorylation compared to total Akt abundance presented as fold from WT. All data are presented as mean±SEM; female mice n=11 WT & n=11 KO and male mice n=12 WT & n=11 KO. FM = fat mass, LM = lean mass. A-H: Data were analyzed using repeated measures mixed effects model where # $p<0.05$ for genotype x time interaction. I&J were analysed by ANOVA with Fishers Post-Hoc test.

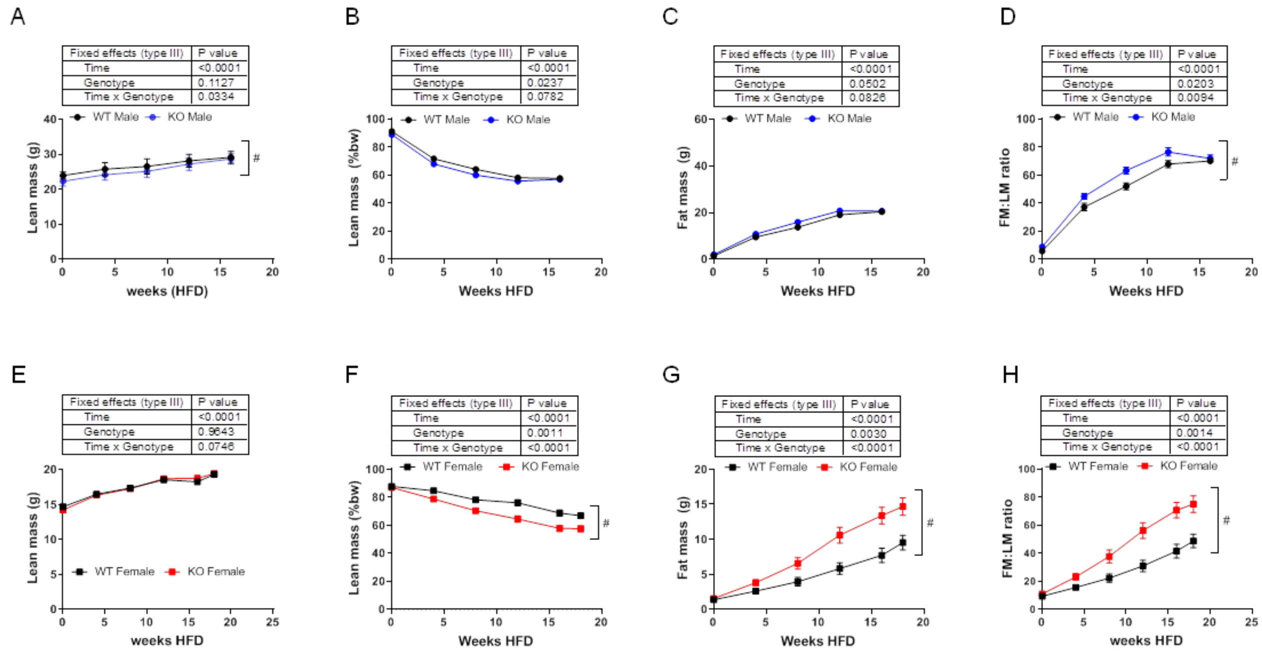

Supplementary Figure 2: Body composition in HFD fed mice. Lean mass in male mice (A) in grams, and (B) lean mass as a percent of bodyweight (%BW). Fat mass in male mice (C) in grams and (D) fat mass to lean mass ratio (FM:LM). Lean mass in female mice (E) in grams, and (F) lean mass as a percent of bodyweight. Fat mass in female mice (G) in grams and (H) fat mass to lean mass ratio. All data are presented as mean±SEM; female mice n=11 WT & n=11 KO and male mice n=12 WT & n=11 KO. FM = fat mass, LM = lean mass. A-H: Data were analyzed using repeated measures mixed effects model where # $p<0.05$ for genotype x time interaction. FM = fat mass, LM = lean mass.

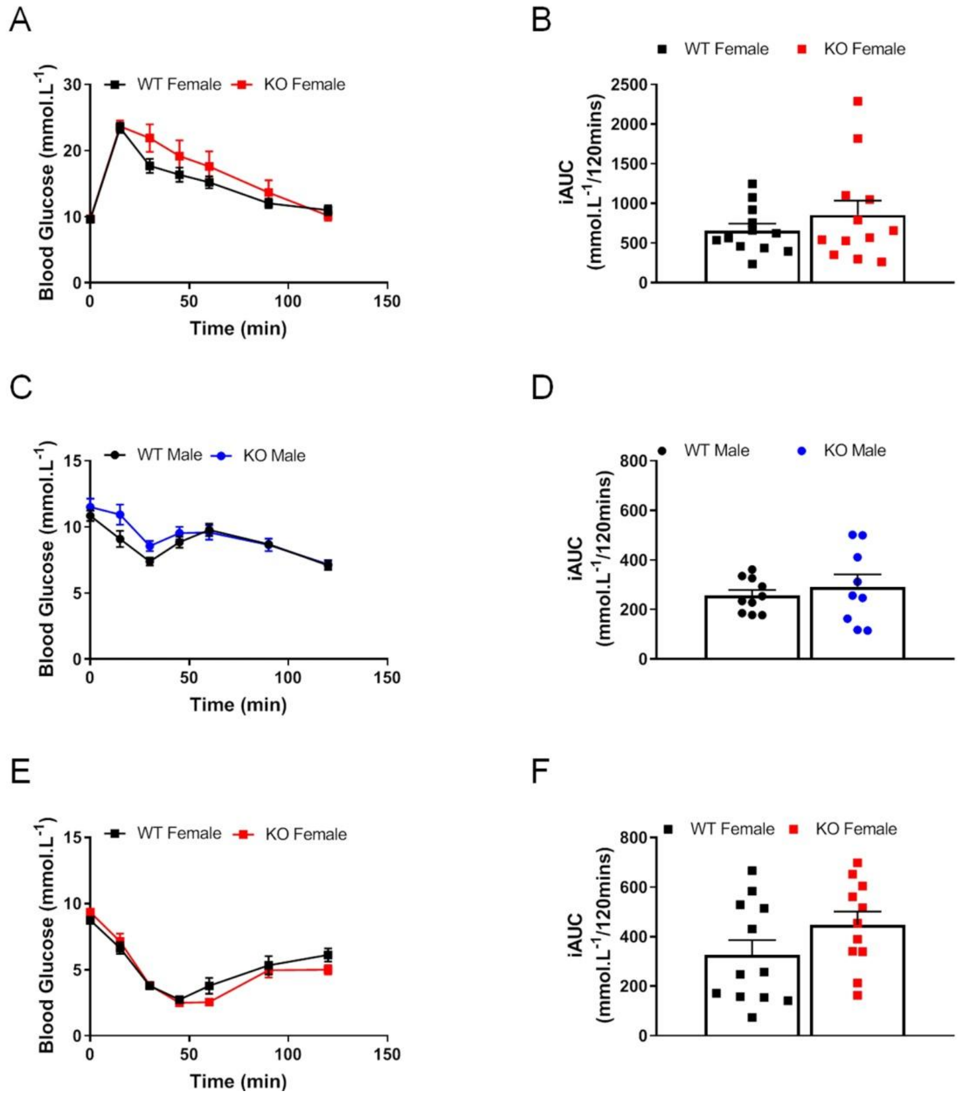

Supplementary Figure 3: Glucose and insulin tolerance tests in male and female mice fed a HFD. (A) Oral glucose tolerance test (oGTT) with glucose dose based on lean mass in WT and Trim28 adi-KO female mice after 18 weeks of HFD, with (B) corresponding incremental area under the curve (iAUC). Intraperitoneal insulin tolerance test (ITT) after 12 weeks of HFD in (C) male and (E) female mice, with corresponding iAUC for (D) male and (F) female mice. All data are presented as mean±SEM, female mice n=12 WT & n=12 KO and male mice n=11 WT & n=9 KO.

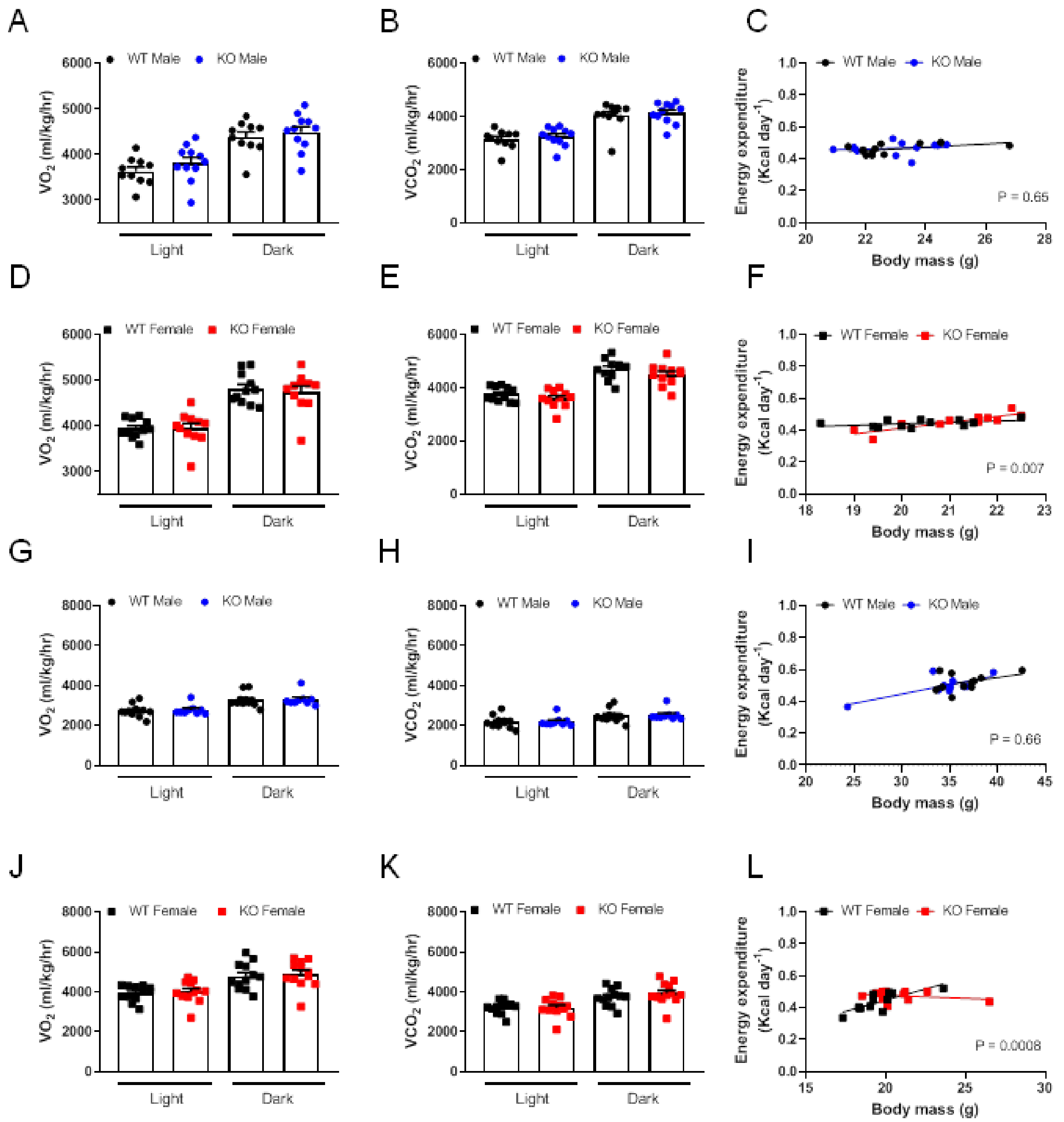

Supplementary Figure 4: Respiratory analysis in chow and HFD fed mice. WT and Trim28 adi-KO VO_2, VCO_2, and energy expenditure ANCOVA adjusted to total body mass in (A-C) chow fed male mice, n=12 WT & n=11 KO; (D-F) chow fed female mice, mice n=11 WT & n=11 KO; (G-I) HFD fed male mice, n=11 WT & n=9 KO; (J-L) HFD fed female mice, n=12 WT & n=12 KO. All data are presented as mean±SEM. All data were analysed by ANOVA with Fisher's LSD post-hoc analysis, except C,F,I&L, which were analysed by Analysis of Co-Variance (ANCOVA).

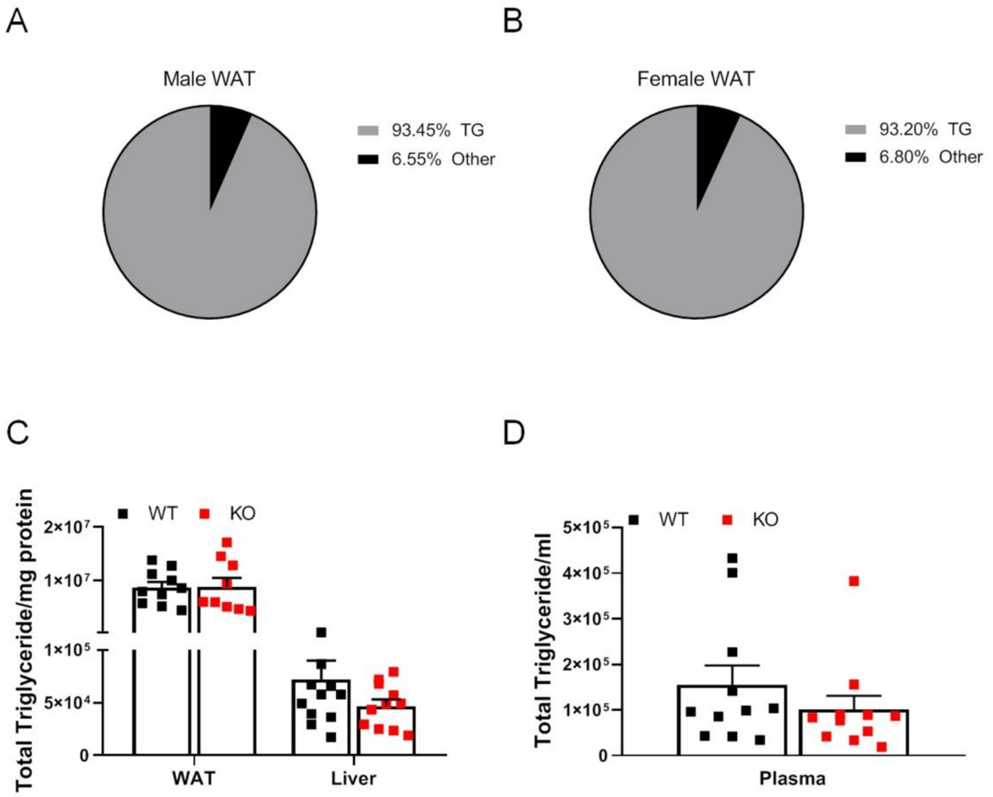

Supplementary Figure 5: Total triglyceride analysis in WAT, liver and plasma. Proportion of total triglycerides in WAT compared with other lipid species as measured by lipidomics analysis for (A) male WAT and (B) female WAT. (C) Total triglycerides (TGs) adjusted to milligrams (mg) of protein in the liver and gonadal fat (WAT) (WAT, n=10 WT and n=9 KO; Liver, n=12 WT and n=11 KO); (D) total TGs per milliliter (mL) of plasma in female wild type (WT) and Trim28 adi-KO (KO) mice fed a chow diet for 24 weeks (n=11/group). Data in C&D are presented as mean±SEM.

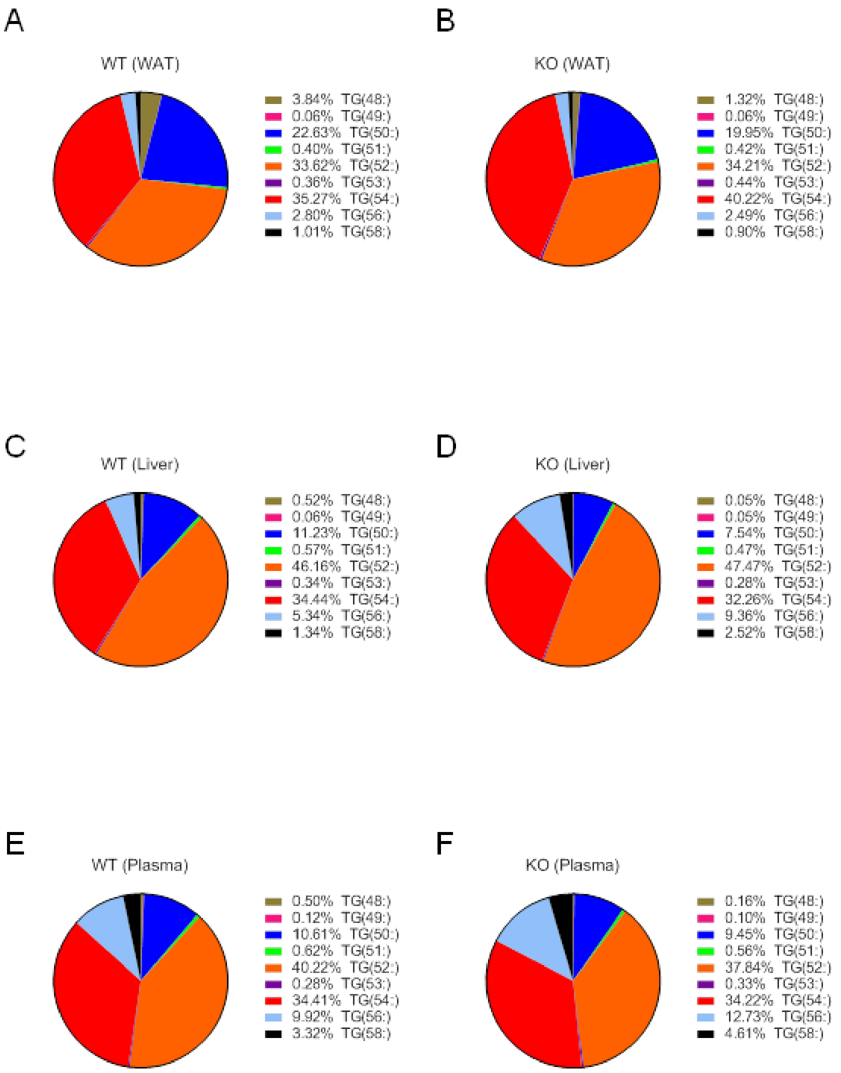

Supplementary Figure 6: Triglyceride species abundance in WAT, liver and plasma. Abundance of triglyceride subclasses measured in single ion monitoring (SIM) mode designated by total FA carbon length, presented as a percent of total triglyceride content in female Trim28 WT and adi-KO (KO) mice fed a chow diet for 24 weeks. Pie charts demonstrating TG class species abundance in (A) WT WAT and (B) KO WAT, (C) WT Liver and (D) KO liver, and (E) WT plasma and (F) KO plasma.

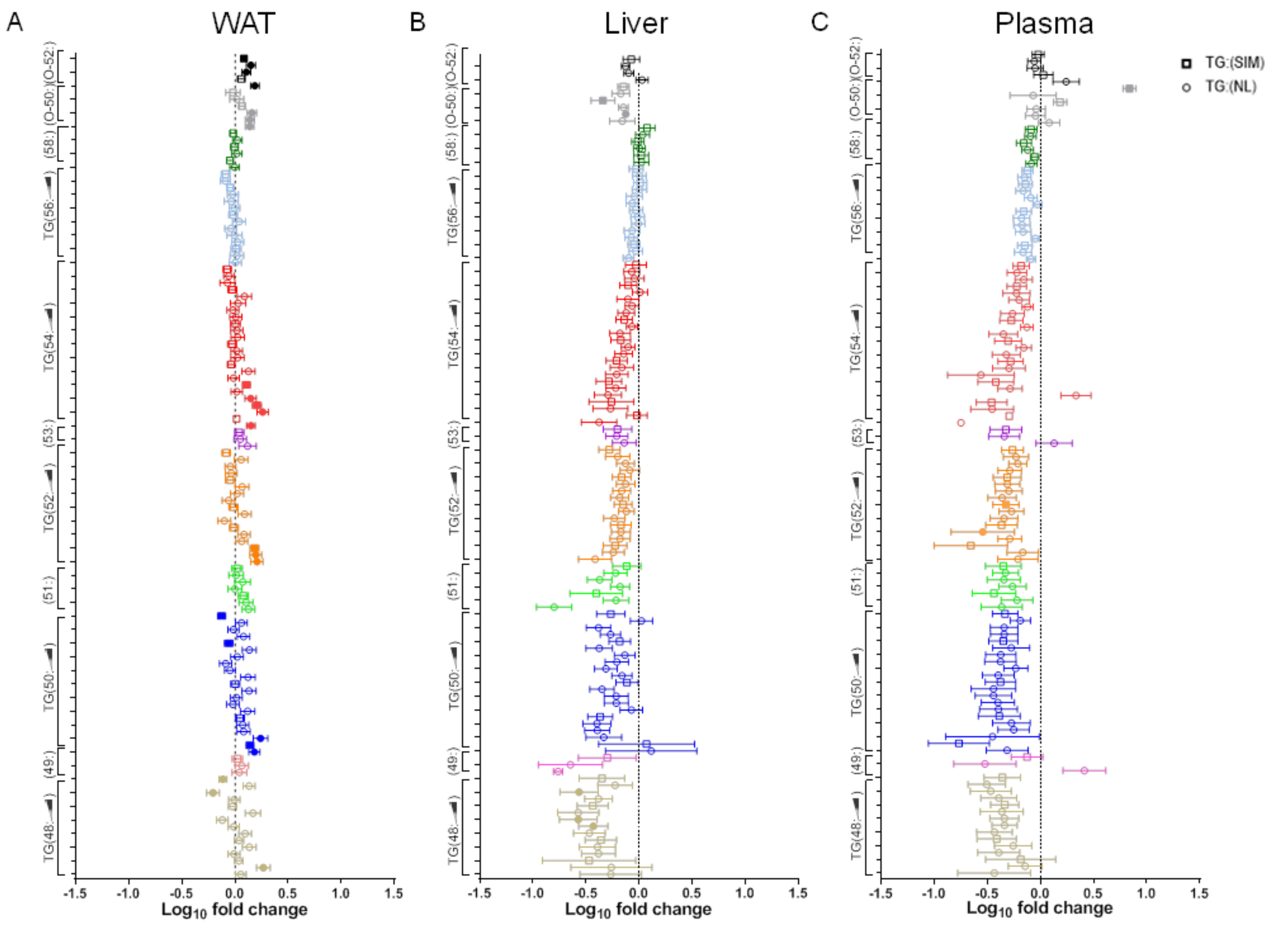

Supplementary Figure 7: Triglyceride composition in WAT of male adipose-specific Trim28 KO mice. Lipidomics in liver, plasma and gonadal fat pads (WAT) from male mice fed a chow diet for 24 weeks. Triglyceride (TG) species from (A) WAT, (B) liver and (C) plasma. Triglyceride species were ordered into sub-classes based on the combined FA carbon chain length (i.e. TG48 = 3 x FAs containing 16C each), then sorted vertically within those sub-classes according to increasing carbon bond saturation (in the order indicated by the scale bar). For a complete list of triglycerides analysed see Supplementary Data Table 1. Squares represent data for single ion monitoring mode (SIM) of lipid species; circles represent species with annotation of a single FA side chain as determined by neutral loss analysis (NL). Data are expressed as Log10 fold change from Trim28 WT mice as mean±SEM, n=12 WT & n=11 KO. Closed symbols represent species that were statistically significant ($p<0.05$) compared to WT, as analysed by Students' 2-way t-test. TG = triacylglycerol, NL = neutral loss, O-TG = alkyl triacylglycerols.

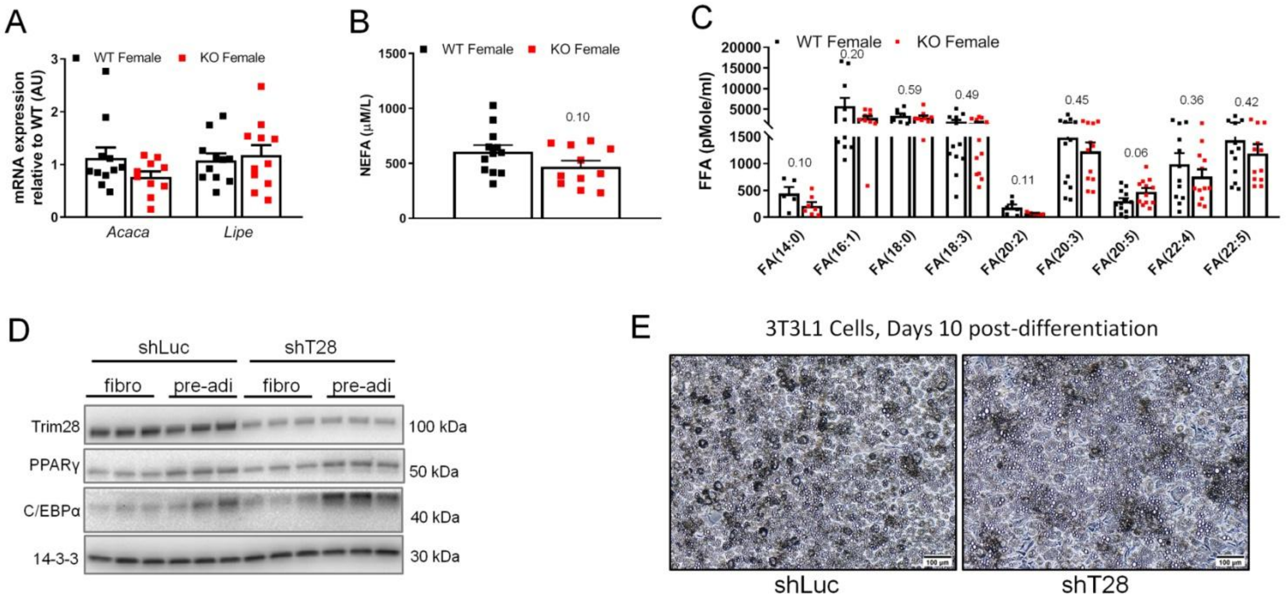

Supplementary Figure 8: Free Fatty acid analysis in mouse plasma and basal characteristics of Trim28 knock-down 3T3-L1 adipocytes. (A) qPCR determined mRNA expression of *Acaca* and *Lipe* genes in white adipose tissue (WAT) (all n=11/group except n=10 for WT *Lipe*). (B) Total NEFA abundance (n=12 WT and n=11 KO) and (C) Lipidomics analysis for low abundance free fatty acids (FFA) in plasma from fasted female HFD fed Trim28 adi-WT and adi-KO mice (FA(14:0), FA(20:2); n=6 WT and n=7 KO, FA(18:3), FA(20:3), FA(20:5), FA(22:4), FA(22:5); n=12/group, FA(16:1), FA(18:0); n=7 WT and n=9 KO). (D) Western blot for Trim28, PPARγ, C/EBPα and pan 14-3-3 in fibroblastic (fibro) and pre-adipocytes (pre-adi) 3T3-L1 cells treated with a control shRNA (shLuc) or shRNA against Trim28 (shTrim28). (E) Photomicrograph of shLuc and shTrim28 3T3-L1 cells 10-days post-differentiation. Cell culture experiments repeated in at least three different independent experiments. Numbers above bars in panel B&C represent p-values between WT and KO mice. All data are mean±SEM. A-C were analysed by ANOVA with Fisher's LSD post-hoc testing.

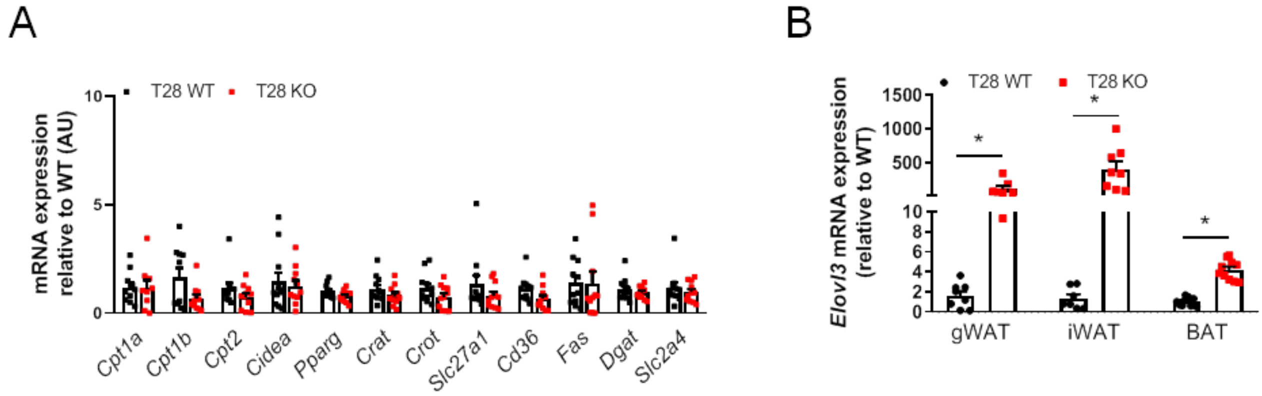

Supplementary Figure 9: *Elovl3* gene expression in WAT and BAT. qPCR gene analysis from WT Trim28 and adi-KO mice for expression of (A) key lipid metabolism genes in gonadal WAT, and (B) *Elovl3* mRNA expression in different fat depots (n=8-10 WT & n=8-10 KO). All data are presented as mean±SEM, * $p<0.05$ versus WT mice. gWAT = gonadal white adipose tissue, iWAT = inguinal white adipose tissue, BAT = brown adipose tissue. Data were analysed by ANOVA with Fisher's LSD post-hoc testing.

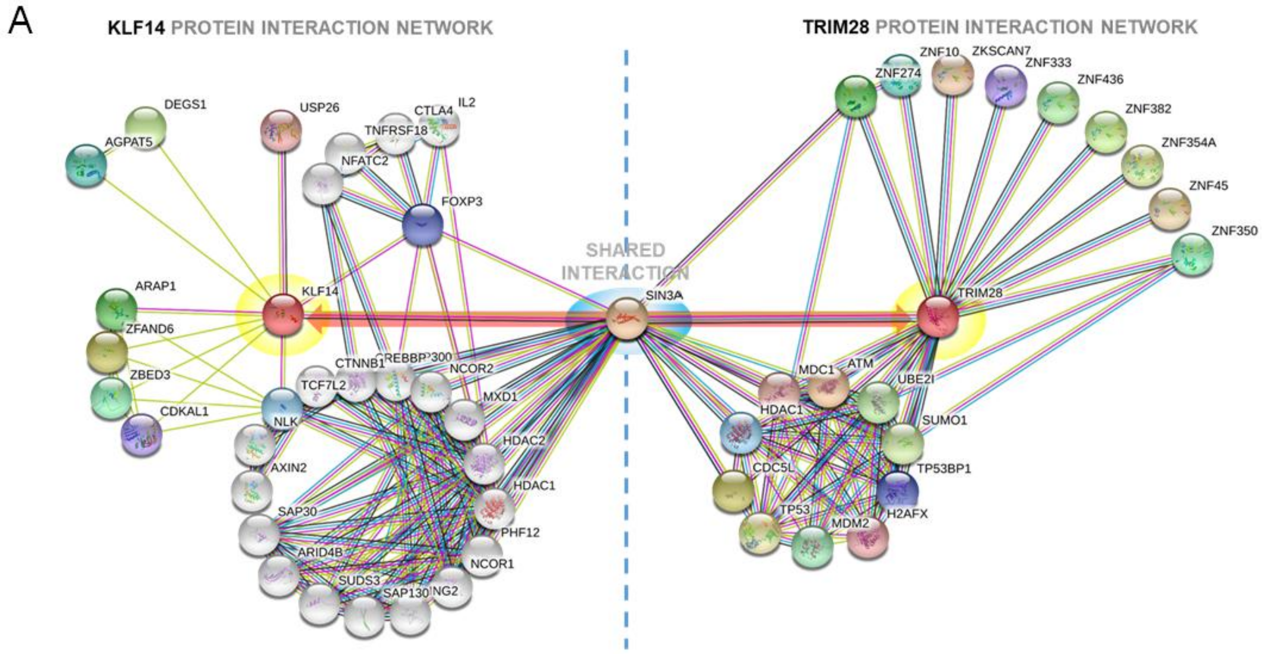

Supplementary Figure 10: STRING Network for KLF14 and TRIM28 protein-protein interactions (PPI). (A) Network of known and manually enriched PPIs for KLF14 (left) and TRIM28 (right) in human cells (separated by blue dotted line) as defined in the method section and presented in detail in Supplementary Data Table 4. A strong enrichment for the HDAC repressor complexes is observed in both networks (cluster at bottom of each network). Both clusters converge though a shared interaction with SIN3A, with the red arrow highlighting the potential co-interaction between KLF14 and TRIM28 via SIN3A.
