# Supplementary material for: Deletion of Trim28 in committed adipocytes promotes obesity but preserves glucose tolerance
Source: Nat Commun. 2021 Jan 4;12:74. doi: 10.1038/s41467-020-20434-3 (PMC7782476; doi:10.1038/s41467-020-20434-3)
Supplement: Supplementary file 3 — Description of Additional Supplementary Files [file 41467_2020_20434_MOESM3_ESM.pdf]

Description of Additional Supplementary Files

File Name: Supplementary Data 1

Description: This file contains the naming format used for TG species quantified (tab1) in our lipidomics analysis. The remaining tabs (2-7) provide the quantified value (umol/mg) of each individual lipid species and/or total lipid class in male and female Epi (WAT), Liver and plasma.

File Name: Supplementary Data 2

Description: This file contains all genes from the RNA-seq analysis of Male (tab1) and female (tab2) WAT presented as fold change compared to WT, and the adjusted (Benjamini and Hochberg) p-value as analysed by DESeq2.

File Name: Supplementary Data 3

Description: qPCR primer sets and the specific sequence (5' to 3') of each forward and reverse oligo for genes analysed in tissues of WT and adi KO mice.

File Name: Supplementary Data 4

Description: Raw data from STRING analysis of KLF14 and TRIM28 interactions as shown in Supplementary Figure 10. Nodes equal first and second level interactions (1 & 2 respectively). Tab 3 = annotation of all nodes combined, with the removal of duplicate (dup) nodes, so that each node only exists once in each dataset. Pink cells in node comparisons are matching proteins from each comparison. Final tab is a summary of the matching proteins from each comparison; shaded cells represent the only proteins that are not in every dataset.
