# Supplementary material for: Deletion of Trim28 in committed adipocytes promotes obesity but preserves glucose tolerance
Source: Nat Commun. 2021 Jan 4;12:74. doi: 10.1038/s41467-020-20434-3 (PMC7782476; doi:10.1038/s41467-020-20434-3)
Supplement: Supplementary file 6 — Supplementary Data 3 [file 41467_2020_20434_MOESM6_ESM.docx]

**Supplementary Table S3.**

Mouse primers for qPCR. (m = mouse).

| **Gene name** | **Primer sequence**  **Forward (5’-3’)** | **Primer sequence**  **Reverse (5’-3’)** |
| --- | --- | --- |
| *mTrim28* | ATTGATCACGGTGCTCTTCC | GTCCTGGACGTCAAAGGGTA |
| *mPpara* | CACGCATGTGAAGGCTGTAA | GCTCCGATCACACTTGTCG |
| *mCox7a* | CAGCGTCATGGTCAGTCTGT | AGAAAACCGTGTGGCAGAGA |
| *mCox8b* | GAACCATGAAGCCAACGACT | GCGAAGTTCACAGTGGTTCC |
| *mPpargc1a* | TGAGGACCGCTAGCAAGTTT | TGAAGTGGTGTAGCGACCAA |
| *mPpargc1b* | TCCTGTAAAAGCCCGGAGTAT | GCTCTGGTAGGGGCAGTGA |
| *mUcp1* | ACTGCCACACCTCCAGTCATT | CTTTGCCTCACTCAGGATTGG |
| *mElovl3* | CCAACAACGATGAGCAACAG | CGGGTTAAAAATGGACCTGA |
| *mLipe* | GCGCTGGAGGAGTGTTTTT | CCGCTCTCCAGTTGAACC |
| *mAcaca* | CTGAAGCAGATCCGCAGCTT | GGTGAGATGTGCTGGGTCATG |
| *mPeg3* | TTTGGAGACAACTGGCAAGA | TCAACTGATCTCCCCTTGCT |
| *mNnat* | AGAACTGCTCATCATCGGCT | TTCGAAAAGCGAATCCTACC |
| *mPark7* | AACACACCCACTGGCTAAGG | GTGCCTCCACAATGGCTAGT |
| *mRplp0* | CTGATAAAGACTGGAGACAAGG | GCTGTAGATGCTGCCATT |
